# Editorial: Progress in Reproductive Neuroendocrinology in Vertebrates

**DOI:** 10.3389/fendo.2019.00895

**Published:** 2019-12-19

**Authors:** Kazuyoshi Tsutsui, Takayoshi Ubuka

**Affiliations:** Laboratory of Integrative Brain Sciences, Department of Biology, Center for Medical Life Science, Waseda University, Tokyo, Japan

**Keywords:** gonadotropin-releasing hormone, gonadotropin-inhibitory hormone, kisspeptin, hypothalamus, pituitary, seasonal reproduction, social information, puberty

The hypothalamic-pituitary-gonadal (HPG) axis is imperative in the control of reproduction in vertebrates. It was thought that gonadotropin-releasing hormone (GnRH) was the only hypothalamic neuropeptide that regulates the HPG axis since its discovery at the beginning of the 1970s (1, 2). However, two new key hypothalamic neuropeptides, i.e., gonadotropin-inhibitory hormone (GnIH) and kisspeptin, have been found in the beginning of the 2000s to play key roles in the control of reproduction (3–6). In 2000, GnIH was discovered in the quail hypothalamus (3). Following intensive researches showed that GnIH inhibits gonadotropin synthesis and release through actions on GnRH neurons and gonadotropes via a G-protein coupled receptor (GPCR), GPR147, in birds and mammals (7). GnIH peptides were also identified in other vertebrate species from fish to humans. As in birds, mammalian and fish GnIH peptides inhibit gonadotropin release, indicating the conserved inhibitory role of GnIH in the regulation of the HPG axis (7). Following the discovery of GnIH, kisspeptin, encoded by the Kiss1 gene, was discovered in mammals. In contrast to GnIH, kisspeptin has a stimulatory effect on GnRH neurons via another GPCR, GPR54 (5, 6). The Kiss1 gene was also identified in amphibians and fish (8). Therefore, we now know that GnRH is not the only hypothalamic neuropeptide controlling reproduction in vertebrates. The aim of this flagship Research Topic is to review the discoveries of GnIH and kisspeptin and the progress in reproductive neuroendocrinology made by these hypothalamic neuropeptides by collecting review articles from leading scientists in this new research field.

The first review article by Tsutsui and Ubuka summarizes the discovery of GnIH and progresses of GnIH research. GnIH was isolated and its structure was determined in 2000 (3). Its function that inhibits gonadotropin release was shown in quail *in vitro* (3) and *in vivo* (4). The article introduces that GnIH inhibits gonadotropin synthesis and release from gonadotropes by acting on gonadotropes and GnRH neurons via GPR147 (9, 10). The article also reviews that GnIH acts in the brain to regulate various behaviors (11–13). The second review article by Son et al. describes the molecular mechanisms of GnIH actions in target cells and how GnIH expression is regulated. Based on the morphology of GnIH neuronal fibers and GnIH receptor, GnRH neurons and gonadotropes are the major targets of GnIH action (3, 14–17). It was demonstrated that GnIH inhibits the adenylate cyclase (AC)/cAMP/protein kinase A (PKA)-dependent pathway both in GnRH neurons and gonadotropes (9, 10). The article further summarizes the mechanisms of how GnIH expression is regulated by glucocorticoid (18) and thyroid hormone (19). The third review article by Angelopoulou et al. introduces that RFRP-3, mammalian GnIH, is involved in the central control of daily and seasonal rhythms of reproduction to synchronize reproductive activity to environmental challenges. Melatonin and thyroid hormones may play critical roles in the regulation of GnIH neurons that convey environmental information to GnRH neurons and gonadotropes (17, 20–23). The fourth review article by Tobari and Tsutsui introduces the effects of social information on GnIH in birds (13). The article reviews researches that investigates the changes in the activities of GnIH neuronal system according to social status. The article introduces the pathway after visual perception of a potential mate and the rapid change in gonadotropin levels via the GnIH neuronal system in male birds. The fifth review article by Di Yorio et al. summarizes what are known and unknown about fish GnIH (24, 25). The article emphasizes that teleost is characterized by three round whole genome duplication that could be responsible for the great phenotypic complexity and variability in reproductive strategies and sexual behavior. The fact may also affect the distribution of GnIH cell bodies and fibers and its relationship with GnRH variants. The article proposes that GnIH may have other functions than reproduction or act as an integrator in the reproductive process in teleosts. The last review article by Uenoyama et al. introduces the triggering role of kisspeptin that controls pubertal onset in mammals. Kisspeptin is a potent secretagogue of GnRH secretion therefore its release is fundamental to pubertal increase in GnRH/gonadotropin secretion. It is thought that puberty is timed by an increase in pulsatile GnRH/gonadotropin secretion in mammals. Recent researches suggest that kisspeptin/neurokinin B/dynorphin A (KNDy) neurons in the arcuate nucleus may play an important role in pulsatile GnRH/gonadotropin secretin during pubertal onset (26). The article further suggests that the timing of pubertal onset is controlled by upstream regulators of kisspeptin expression and release.

The review articles collected in this flagship Research Topic acknowledge that GnIH and kisspeptin play important roles in the hypothalamic control of reproduction, which is indispensable in developmental, seasonal, and social regulation of reproductive activities in vertebrates.

## Author Contributions

TU wrote the manuscript. KT edited the manuscript.

### Conflict of Interest

The authors declare that the research was conducted in the absence of any commercial or financial relationships that could be construed as a potential conflict of interest.
